# Case Report: Complete atrioventricular block in an elderly patient with acute pulmonary embolism

**DOI:** 10.3389/fcvm.2024.1355000

**Published:** 2024-02-06

**Authors:** Moojun Kim, Chang-ok Seo, Hangyul Kim, Hye Ree Kim, Kyehwan Kim, Min Gyu Kang, Jeong Rang Park

**Affiliations:** Division of Cardiology, Department of Internal Medicine, Gyeongsang National University Hospital and Gyeongsang National University School of Medicine, Jinju, Republic of Korea

**Keywords:** pulmonary embolism, atrioventricular block, right bundle branch block, left bundle branch block, right ventricle

## Abstract

**Introduction:**

Multiple abnormal electrocardiographic findings have been documented in patients experiencing acute pulmonary embolism. Although sinus tachycardia is the most commonly encountered rhythmic disturbance, subsequent reports have highlighted other findings. These include right bundle branch block, right axis deviation, nonspecific ST segment/T wave changes, and T wave inversion in the right precordial leads. To date, only a limited number of cases involving a complete atrioventricular block have been reported in acute pulmonary embolism.

**Case presentation:**

Here, we present the case of a 91-year-old woman with acute pulmonary embolism, whose initial electrocardiogram showed a complete atrioventricular block. She presented with presyncope and an initial blood pressure of 77/63 mmHg. Echocardiography confirmed signs of right ventricular dysfunction. Catheter-directed thrombolysis and a temporary pacemaker insertion were carried out sequentially. The following day, electrocardiography showed sinus rhythm with a left bundle branch block.

**Discussion:**

The presence of a complete atrioventricular block in patients with acute pulmonary embolism serves as a clinical marker of high-risk status.

## 1 Introduction

Acute pulmonary embolism (PE) is a significant contributor to global mortality, morbidity, and healthcare expenditures. It is a commonly diagnosed clinical entity that has been extensively discussed in the literature, with annual incidence rates ranging from 39 to 115 per 100,000 population (1). The abrupt obstruction of the pulmonary artery system leads to various pathophysiological complications, predisposing patients to develop a sudden increase in pulmonary vascular resistance, resulting in right ventricular dilatation and altering the contractile properties of the RV myocardium via the Frank-Starling mechanism (1).

Since McGinn and White first reported what is now the traditional S1Q3T3 pattern in 1935 (2), sinus tachycardia, incomplete or complete right bundle branch block (RBBB), right axis deviation, nonspecific ST segment/T wave changes, and T wave inversion in the right precordial leads have been investigated for their incidence and prognostic power.

However, the incidence of complete atrioventricular block (CAVB) remains rare and noteworthy in patients with PE. Only a limited number of cases have been reported in the literature (3–8). Herein, we present the case of a 91-year-old woman who presented with CAVB and review the relevant literature.

## 2 Case presentation

A 91-year-old woman presented to the emergency room with a sudden, transient loss of consciousness following a sensation of lightheadedness. The patient had a documented medical history of hypertension, osteoporosis, and multiple degenerative changes in the lumbar spine that resulted in prolonged immobility and a sedentary lifestyle.

**Figure 1:**
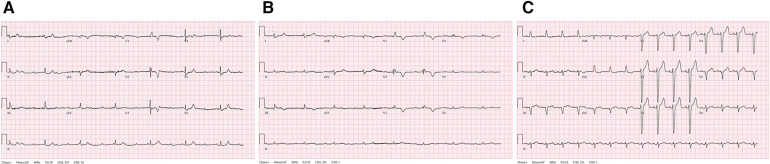
Serial changes of recorded 12-lead electrocardiogram (ECG). (**A**) The initial ECG shows a complete atrioventricular block (CAVB) with right bundle branch block. (**B**) The immediate post-thrombolytic ECG with unresolved CAVB. (**C**) Normal sinus rhythm with left bundle branch block at 88 beats per minute.

Initial vital signs of the patient showed hemodynamic instability, with a blood pressure of 77/63 mmHg, pulse rate of 58 beats per minute, and respiratory rate of 29 beats per minute. The pulse oximeter indicated a saturation of 92% at 5 L/min of oxygen through a non-rebreather mask. The physical examination findings were unremarkable and showed no signs of mental impairment or neurological issues, as evidenced by a Glasgow Coma Scale score of E4M5V6. The initial chest radiography results revealed cardiomegaly and a prominent aortic knob but no other significant abnormalities. The 12-lead ECG recordings indicated CAVB and RBBB with a QRS duration of 124 ms and T-wave inversions in lead V1-V3 (Figure 1A). The patient had no known history of underlying arrhythmias or cardiac problems related to acute changes in hemodynamic collapse.

Laboratory findings showed a hemoglobin level of 7.9 g/dl and a normal platelet count of 251,000/mm^3^. The results of the biochemical tests were as follows: sodium 138.1 mmol/L [reference range (RR): 135–145 mmol/L], potassium 4.1 mmol/L (RR: 3.3–5.1 mmol/L), total CO_2_ 9 mmol/L (RR: 21–30 mmol/L), LDL-cholesterol 98 mg/dl (RR: 0–130 mg/dl), lactic acid 9.40 mmol/L (RR: 0.5–2.2 mmol/L), and creatinine 1.26 mg/dl (RR: 0.5–0.9 mg/dl). The high-sensitivity cardiac troponin T (hs-TnT) level was 117 ng/L, surpassing the upper normal threshold of 14 ng/L. Markedly elevated fibrinogen degradation product (>120 ug/ml; RR: 0–2.01 ug/ml) and D-dimer (>20 ug/ml; RR: 0–0.5 ug/ml) heightened the clinical suspicion of acute PE.

**Figure 2:**
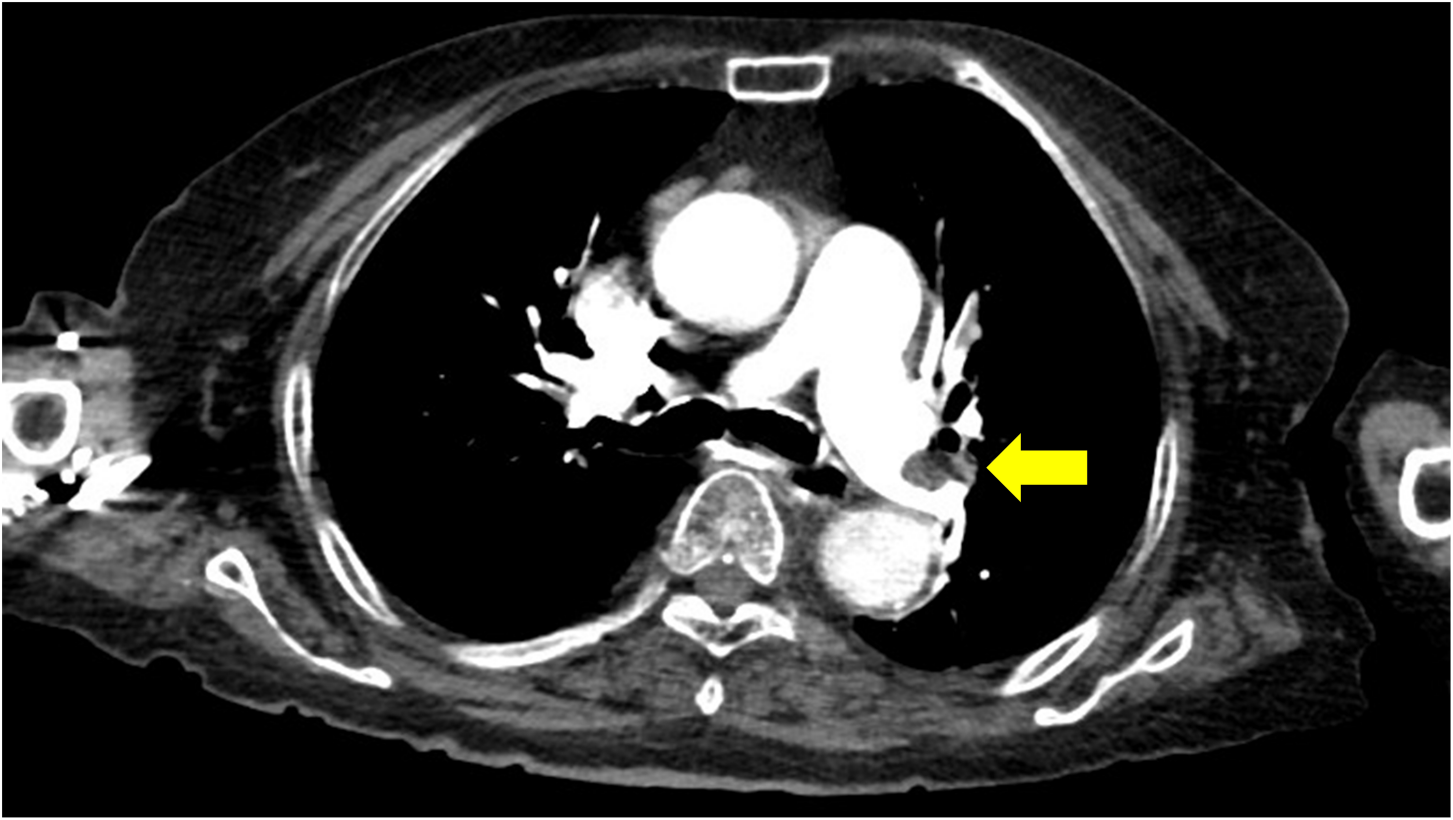
Computed tomographic pulmonary angiography indicating pulmonary emboli in the distal left main pulmonary artery.

Computed tomographic pulmonary angiography (CTPA) revealed an enlarged right ventricle (RV) with multiple filling defects in the distal left main pulmonary artery that extended into both lobar and segmental pulmonary arteries of both lungs (Figure 2). Lower extremity ultrasonography revealed a low-density focal filling defect in the right calf vein, suggesting deep vein thrombosis.

A comprehensive two-dimensional transthoracic echocardiography revealed an enlarged RV with a concomitant D-shaped left ventricle. The RV displayed qualitative dilatation with a diameter exceeding 41 mm at the base of the apical 4-chamber view. Additionally, the patient exhibited akinesia of the basal and mid-portion of the free wall, commonly known as McConnell's sign that is indicative of severe dysfunction (Supplementary Movie S1). RV systolic dysfunction was documented as follows: a tricuspid lateral annular systolic velocity (RV S') of 0.06 m/s, tricuspid annular plane systolic excursion (TAPSE) of 10 mmHg, and fractional area change (FAC) of 27%. Mild tricuspid insufficiency was noted, accompanied by an estimated pulmonary arterial pressure (PAP) of 54 mmHg.

Subsequently, the patient experienced progressive clinical deterioration with a blood pressure of 68/41 mmHg. She was resuscitated with intravenous fluid, norepinephrine administration was initiated, and catheter-directed thrombolysis was performed. Alteplase was infused at 1 mg/h per catheter for 6 h and the patient was continuously monitored in the intensive care unit. Nonetheless, the patient continued to report persistent chest discomfort and shortness of breath, along with wheezing, and demonstrated fluctuations in oxygen saturation within the range of 70%–80%. The ECG results still indicated CAVB with complete RBBB (Figure 1B) and RV dysfunction persisted as documented via echocardiography. Considering the persistent symptoms and remaining signs of high-risk acute PE even after thrombolytic therapy, we decided to insert a temporary transvenous pacemaker.

The following day, the ECG results of the patient showed a return to a normal sinus rhythm, and subsequent echocardiography showed markedly improved RV function: RV S' 0.10 m/s, TAPSE 17 mm, and FAC 46% (Supplementary Movie S2). The estimated PAP was decreased to 35 mmHg. Within 72 h, her intrinsic sinus rhythm was fully restored, and the temporary pacemaker was removed. A subsequent ECG revealed the presence of a baseline left bundle branch block (LBBB) that had not been documented previously (Figure 1C). The patient had gained enough mobility to ambulate independently by the time of discharge and has been undergoing anticoagulation therapy since that time.

## 3 Discussion

Patients with acute PE exhibit considerable variability in ECG abnormalities that are of relatively minor importance in the diagnostic process because they lack the sensitivity and specificity required to definitively diagnose or exclude PE. It has been reported that approximately 10%–25% of patients with PE have a completely normal ECG pattern (9), and the commonly observed abnormal ECG changes include RBBB, rightward or leftward shift of the frontal plane QRS axis, clockwise rotation and shift of the transition zone, a classic S1Q3T3 pattern of acute cor pulmonale, nonspecific ST segment/T wave changes, T wave inversions in the right precordial leads, and atrial arrhythmias (9–11). Some rare findings described in case reports include electrical alternans (12) and ST-segment elevation in lead aVR, with ST-segment depression in leads I and V4-V6 (13). Specifically, the RBBB has been identified as an indicator of RV strain found in more severe cases of PE, with reported incidences ranging from 6% to 67% (8). However, the prognostic significance of an ECG result indicating RV strain remains inconclusive (14, 15).

To our knowledge, the simultaneous occurrence of syncope and CAVB in patients with massive PE is rare, with only a few cases documented in the literature. In 1983, Wilner et al. first documented two cases of PE accompanied by syncope in patients with preexisting LBBB (5). The syncope episodes were not attributable to typical cardiovascular collapse, but instead to a paroxysmal atrioventricular (AV) block. They proposed that the right branch of the His bundle was particularly exposed to its superficial subendocardial trajectory on the RV side of the septum, making it highly vulnerable to a sudden distension of the right cavities. Martí et al. (6). Mukerji et al. (7) reported a complete AV block secondary to PE in two patients who had LBBB on admission. The widely accepted mechanism through which PE induces conduction disturbance is as follows: a rapid elevation in RV pressure that results from obstruction of the pulmonary artery system triggers an acute RV volume overload. This overload induces compression of the intraventricular septum, giving rise to the RBBB. Herein the RBBB, primarily attributed to a mechanical phenomenon in conjunction with the LBBB, resulted in CAVB and subsequent hemodynamic instability. This acute overload state was also responsible for RV dysfunction findings that were qualitatively measured using echocardiography.

Another potential mechanism involves embolic events that trigger myocardial ischemia in the AV node, resulting in CAVB. Although we did not perform invasive coronary angiography to rule out the presence of coronary artery disease conclusively, the absence of definitive left ventricular wall motion abnormalities detectable via echocardiography and the substantial reduction in serial hs-TnT levels suggest that acute myocardial ischemia may not have been the primary contributor in this case.

In this specific case, one possible pathophysiological factor is the susceptibility to or fragility of conduction disturbances in the elderly population. Previous epidemiologic studies (16, 17) have shown that older age is a risk factor for CAVB. However, in our case, we have highlighted the correlation between RV dysfunction and ECG changes, as well as the observed dissociation of AV and subsequent restoration of conduction, all confirmed through echocardiography. While older age may indeed contribute as a factor influencing CAVB, we believe that RV dysfunction plays a more pivotal role in this particular case.

There is an ongoing debate regarding the association between the RBBB and mortality in PE (14, 15). However, Keller et al. (14) reported that RBBB- and S1QIII-type patterns were associated with RV overload and cardiac injury. RV dysfunction detectable via echocardiography and increased levels of cardiac biomarkers of myocardial ischemia are well-established prognostic markers of acute PE.

It is noteworthy that the uncommon nature of this clinical presentation, involving simultaneous CAVB and PE, may substantially contribute to delayed or even missed diagnoses of this syndrome (18, 19). In our case, the degree of RV dysfunction ameliorated rapidly after thrombolytic therapy, occurring simultaneously with the disappearance of CAVB and confirmation of sinus conversion. Therefore, we reason that CAVB is likely a consequence of acute RV strain linked to the underlying LBBB. We speculate that the presence of RBBB and/or CAVB in patients with acute PE should be considered a robust indication for thrombolytic therapy.

In conclusion, given the uncommon nature of this clinical scenario, clinicians should be mindful of the close association between CAVB and PE, particularly in patients with preexisting LBBB. We propose that CAVB can serve as an early indicator of a high-risk status in acute PE. Therefore, in similar situations, a prompt diagnosis of PE and timely treatment strategies should be applicable.

## Data Availability

The original contributions presented in the study are included in the article/Supplementary Material, further inquiries can be directed to the corresponding author.

## Supplementary material

Supplementary Movie S1 Initial echocardiography revealed right atrial and ventricular dilatation with akinesia of the basal and mid-portions of the right ventricular free wall or McConnell’s sign.

Supplementary Movie S2 Follow-up echocardiography at the time of sinus conversion showed improved right ventricular dilatation, systolic function, and dyskinetic septal motion from the left bundle branch block.
